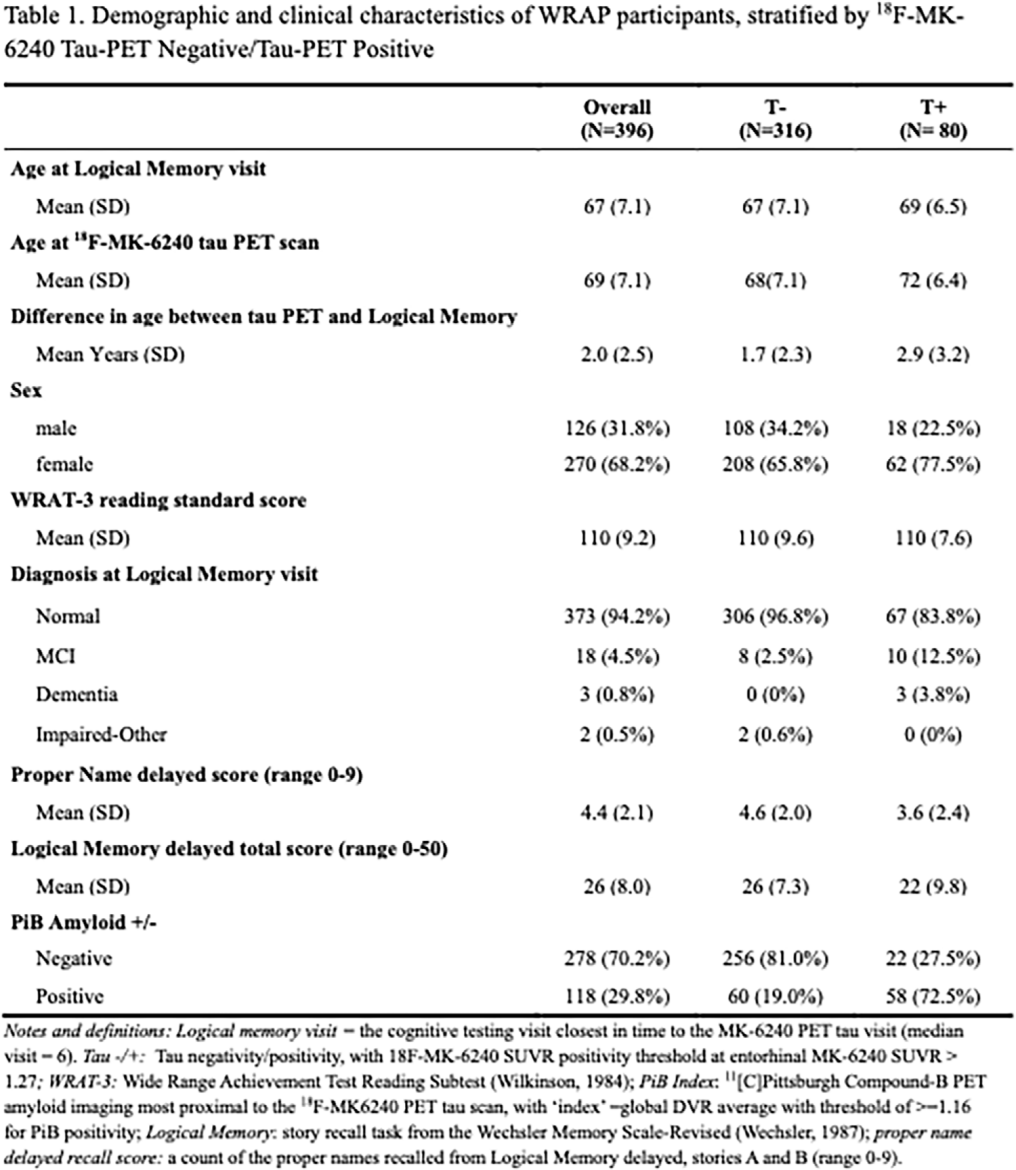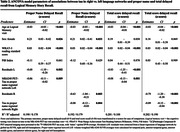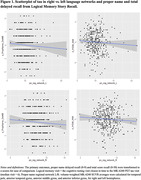# The relationship between right and left hemisphere regional tau and proper name recall from Logical Memory story recall

**DOI:** 10.1002/alz.085701

**Published:** 2025-01-03

**Authors:** Hayley A Olson, Rebecca E. Langhough, Davide Bruno, Bruce P Hermann, Kristin E Basche, Beryl Miess, Noelle Kaminski, Olivia Mandel, Bradley T. Christian, Sterling C. Johnson, Tobey J. Betthauser, Kimberly D Mueller

**Affiliations:** ^1^ University of Wisconsin‐ Madison, Madison, WI USA; ^2^ University of Wisconsin‐Madison, Department of Medicine, Madison, WI USA; ^3^ Liverpool John Moores University, Liverpool United Kingdom; ^4^ School of Medicine and Public Health, University of Wisconsin‐Madison, Madison, WI USA; ^5^ University of Wisconsin‐Madison School of Medicine and Public Health, Madison, WI USA; ^6^ University of Wisconsin‐Madison, Madison, WI USA; ^7^ Geriatric Research Education and Clinical Center, William S. Middleton Memorial Veterans Hospital, Madison, WI USA; ^8^ Department of Medicine, University of Wisconsin‐Madison School of Medicine and Public Health, Madison, WI USA; ^9^ Department of Communication Sciences and Disorders, University of Wisconsin‐Madison, Madison, WI USA

## Abstract

**Background:**

Sensitive screening for early Alzheimer’s disease (AD)‐related cognitive decline are needed. Prior research links high beta‐amyloid (Aβ) levels to reduced proper name (PN) retrieval in individuals without cognitive impairment. We examined whether language‐related regional tau from PET associated with Logical Memory (LM) proper name recall, accounting for LM covariates.

**Method:**

Participants included n = 396 from the Wisconsin Registry for Alzheimer’s Prevention (WRAP) with Logical Memory (LM) story recall, amyloid PET ([11C]Pittsburgh Compound‐B, PiB), and [18F]‐MK‐6240 tau PET; all were unimpaired at LM baseline. Outcomes included z‐scores for LM delayed total score (“total‐LM”) and PN delayed LM subscore (“PN‐score”) from the visit most adjacent to PET‐tau imaging. Amyloid PET burden (visit adjacent to tau PET) was summarized as a global PIB DVR (PiB index, cerebellum GM reference region, graphical analysis). Left and right volume‐weighted MK‐6240 SUVR (inferior cerebellar GM reference regions) averages were calculated for regions previously associated with PN retrieval, including temporal pole, anterior temporal gyrus, anterior middle gyrus, and anterior inferior gyrus (“PN‐regional‐tau‐network”). We compared results from two ANCOVA models (L vs. R regional network as predictors) for each outcome (PN‐score, total‐LM). Covariates in each model included age at LM, sex, WRAT‐3 reading, and PiB index (to replicate prior analyses). To account for unexplained variance from global tau in entorhinal cortex, residuals from a regression of global tau on PN‐regional‐tau‐network were also included.

**Result:**

Table 1 displays demographics overall and by tau status (T+/T‐). Overall mean age was 67 (SD = 7); n(%) = 80(20.2%) were T+. Left and right PN‐regional‐tau‐networks were significant predictors of PN‐score (β = ‐.06, R^2^ = .190, β = ‐.08, R^2^ = .192, respectively) and total‐LM (β = ‐.08, R^2^ = .201, β = ‐.09, R^2^ = .202, respectively; Table 2, Fig 1). In sensitivity analyses removing n = 23 participants with MCI or dementia, Right‐hemisphere PN‐regional‐tau (but not left) was still a significant predictor of PN‐score (β = ‐.06, R^2^ = .190), and L and R PN‐regional‐tau significantly predicted total‐LM (β = ‐.06, R^2^ = .134, β = ‐.07, R^2^ = .136, respectively).

**Conclusion:**

Logical Memory story recall measures may be sensitive to tau burden in specific language regions; right hemisphere tau in the PN‐regional‐network may be particularly sensitive to early tau burden. Future directions include characterizing regional PET patterns and plasma‐biomarker‐related preclinical rates of change in these outcomes.